# An Effective Liposome-Based Nanodelivery System for Naphthalene Derivative Polyamines with Antitumor Activity

**DOI:** 10.3390/biom14111347

**Published:** 2024-10-23

**Authors:** Isabel Pont, Rubén Felipe, Juan C. Frías, Javier U. Chicote, Antonio García-España, Enrique García-España, M. Teresa Albelda

**Affiliations:** 1 Instituto de Ciencia Molecular, Departamento de Química Inorgánica, Universidad de Valencia, 46010 Valencia, Spain; isabel.pont@uv.es (I.P.); rufedo@alumni.uv.es (R.F.); enrique.garcia-es@uv.es (E.G.-E.); 2 Departamento de Ciencias Biomédicas, Universidad Cardenal Herrera-CEU, CEU Universities, 46115 Alfara del Patriarca, Spain; juan.frias@uchceu.es; 3 Unitat de Recerca, Hospital Joan XXIII, Institut de Investigació Sanitaria Pere Virgili (IISPV), Universitat Roviri i Virgili, 43002 Tarragona, Spain; antoniogem85@gmail.com; 4 Department of Inorganic Chemistry, University of Valencia, 46010 Burjassot, Spain

**Keywords:** liposomes, polyamines, cytotoxicity, cancer cells

## Abstract

This study focuses on the development of a novel liposome-based nanodelivery system designed to encapsulate polyamine-1, a compound with potential anti-tumor properties. The main objective of this work was to enhance the therapeutic and imaging potential of polyamine-1 by incorporating it into liposome-based nanoparticles, which were functionalized with a gadolinium complex for imaging purposes and a fluorescent phospholipid for tracking applications. These nanoparticles were characterized by measuring their size, shape, polydispersity index, zeta potential and encapsulation efficiency. In vitro experiments were conducted to evaluate the antitumor activity, specifically determining the cytotoxicity of both free and encapsulated polyamine-1 in cancerous and non-cancerous cell lines. Additionally, the study shows the enhanced signal intensity of gadolinium-loaded liposomes by T1-weighted MRI, highlighting their imaging potential. The experimental results suggest that this liposome-based nanodelivery system not only has therapeutic potential in targeted cancer therapy but also could be advantageous for diagnostic imaging, particularly in MRI applications.

## 1. Introduction

Cancer is the leading cause of morbidity and mortality worldwide, accounting for nearly 10 million deaths in 2022 [1]. The incidence of this disease is increasing rapidly; there were an estimated 14.1 million cancer cases worldwide in 2012, and an estimated 35 million new cancer cases are expected by 2050 [2]. Advances in treatment and early detection have been crucial to improve survival for patients [3,4,5,6]. But, since the majority of tumors are heterogeneous and many cancers contain a small population of cells highly tumorigenic and intrinsically drug-resistant, widely used treatments such as chemotherapy require the use of relatively high doses of anti-cancer drugs, which produces an unwanted strong non-specific toxicity [7,8]. Thus, targeted therapies have emerged as one of the most promising alternatives to overcome these challenges by enhancing the pharmacological action of anti-cancer drugs and reducing toxic side effects [9].

In parallel, the emergence of small molecules able to interact with specific cell biomolecules essential for the tumor’s growth and progression has fostered targeted therapies [10].

Natural polyamines (putrescine, spermidine and spermine) are polycationic compounds at physiological pH with proven capacity to modulate a large variety of biochemical processes like signaling, cell proliferation, gene expression, enzyme activity, activation of DNA synthesis, protein–DNA interactions and ion channel function [11,12,13]. Dysregulation of polyamines has been implicated in cancer progression, as elevated levels of these compounds have been detected in various tumors. Specifically, increased polyamine levels have been associated with several types of cancer, including breast, colon, prostate and skin cancers [14,15,16]. Moreover, polyamines have been shown to interact with DNA, affecting the stability of single-, double- and triple stranded-DNA, and protecting DNA from oxidative stress, damaging agents, ionizing radiation and endonuclease digestion. This selective binding of polyamines to DNA has attracted significant attention in cancer research since this interaction interferes with processes like transcription and replication [17]. Therefore, research efforts have been directed toward the development of polyamine analogues able to target nucleic acids as anticancer drugs [18,19,20]. For instance, the combination of natural polyamines with naphthalimide or 1,4-naphthoquinone has been shown to enhance interaction with DNA, contributing to their antitumor efficacy [21]. Furthermore, attaching a substituted spermidine tail to the naphthoquinone core of lapachol resulted in a remarkable increase in selective cytotoxicity for glioblastoma cells while preserving healthy astrocytes [22]. Chalcone–polyamine conjugates exhibited selective cytotoxic effects against colorectal and prostatic cancers in various cell lines [23]. Lastly, polyamine–flavonoid conjugates displayed a moderate cytotoxic effect in liver tumor cells [24].

**Scheme 1:**
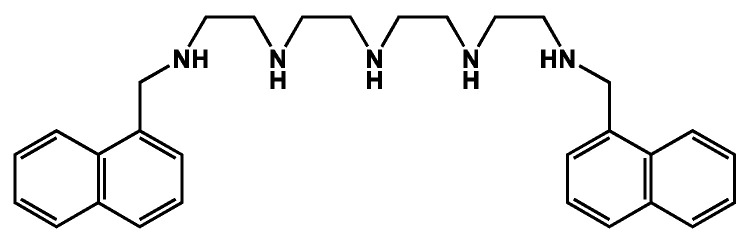
Chemical structure of polyamine-1 (N-naphthalen-1-ylmethyl-N′-[2-(2-(2-[naphthalen-1-ylmethyl) amino]ethylamino]ethylamino)ethyl]ethane-1,2-diamine).

Other cyclic systems, such as acridine conjugates, have been used for cancer treatment since the 1970s due to their ability to intercalate into DNA and inhibit topoisomerase and telomerase, further contributing to their antitumor effects [25]. Moreover, naphthalene derivatives have demonstrated significant potential as inhibitors of topoisomerase, cytoplasmic protein–tyrosine phosphatase and microtubules [26]. Notably, naphthalene-substituted triazole spirodienones have been shown to suppress breast cancer 4T1 tumor growth, indicating that they may be potential anticancer agents for further development [27]. Polyamine conjugates with bis(naphthalene-2-carboxamides) terminal groups exhibited moderate cytotoxicity in prostate carcinoma and mammary gland adenocarcinoma cell lines due to their mild ability to stabilize double helix DNA through stacking interactions [28]. Earlier studies in our group with naphthyl functionalized open-chain polyamines revealed an unusual stabilization of double-stranded polydA–polydT sequences, used as a DNA model, which suggests a bis-intercalative mode of interaction [29]. This effect was particularly strong with the polyamine (N-naphthalen-1-ylmethyl-N′-[2-(2-{2-[(naphthalen-1-ylmethyl) amino] ethylamino} ethylamino) ethyl]ethane-1,2-diamine) (hereafter polyamine-1, see Scheme 1). Due to its high affinity towards DNA and the multiple positive charges, polyamine-1 could be considered as an analogue of spermidine, offering promising potential as a chemotherapeutic agent; however, its antitumor effects have not yet been evaluated in cancer cell lines.

Given the promising interactions of polyamines with DNA and their potential as therapeutic agents, achieving effective cancer treatment requires that these agents selectively target tumor cells while maintaining cytotoxic concentrations [14]. Therefore, it is essential to explore effective delivery systems to enhance their bioavailability and selective targeting in cancer treatment. Nanotechnology has emerged as a promising solution, offering a large variety of nanomaterials to provide delivery approaches able to efficiently carry polyamine derivative drugs to tumoral cells [30].

Lipid-based carriers, particularly liposomes, are recognized among drug-delivery vehicles due to their unique structural properties, favorable safety profile, excellent biocompatibility, minimal toxicity, reduced side effects and favorable risk–benefit ratio [31]. As one of the first discovered self-assembled nanoparticles made from natural biomaterials, liposomes have been extensively studied in various applications, including vaccines, imaging and cosmetics [32]. Liposomes can release drugs upon demand and offer several advantages, including enhanced pharmacokinetics, prolonged circulation time and passive targeting to tumors and inflamed tissues through the enhanced permeability and retention (EPR) effect, while also minimizing systemic toxicity, improving drug solubility and enabling slow, sustained release of encapsulated drugs [33]. Moreover, liposomes can be functionalized to serve as effective imaging agents, complementing their role in drug delivery. By incorporating specific targeting moieties, such as antibodies, peptides or ligands, these modified liposomes can selectively bind to specific cell types or tissues, allowing for precise imaging of tumor sites or disease progression. This dual functionality not only facilitates improved therapeutic delivery but also establishes functionalized liposomes as a powerful tool in both cancer treatment and diagnostic applications [34].

Doxil (liposomal doxorubicin), as one of the most successful liposome formulations, has demonstrated the effectiveness of these vehicles in enhancing drug solubility and targeted delivery, setting a precedent for the broader use of liposomes in therapies [35,36]. The ability of liposomes to encapsulate both lipophilic and hydrophilic drugs highlights their versatility as delivery systems. Although specific studies on polyamine encapsulation in liposomes are limited [37,38], their adaptable nature and success in administering other therapeutic agents make this strategy promising for future research into polyamines and their potential in cancer treatment.

In this study, we report the innovative encapsulation of polyamine-1 (N-naphthalen-1-ylmethyl-N′-[2-(2-{2-[(naphthalen-1-ylmethyl) amino] ethylamino} ethylamino) ethyl] ethane-1,2-diamine) within liposomes. Encapsulating this compound within liposomes would aim not only to enhance its bioavailability but also to facilitate its passage through the cell membrane, as its polycationic nature, being positively charged at physiological pH, would limit its ability to diffuse passively through the hydrophobic lipid bilayer. Additionally, encapsulation could help preserve its structural integrity and affinity for DNA by protecting it from potential interactions with external factors. Previous studies on the interaction of this polyamine with DNA have demonstrated that upon interaction with Cu(II) salts, the compound undergoes significant conformational changes [29]. These changes alter its bis-intercalating mode, leading to the loss of functionality of one of the aromatic groups and reducing the ligand’s overall affinity, shifting from bis-intercalation to intercalation with a single aromatic unit. Encapsulation within liposomes could mitigate these structural distortions, thereby maintaining the compound’s therapeutic potential.

In addition, we present a comprehensive characterization of the resulting nanoparticles, supported by advanced imaging studies. Furthermore, we evaluate the cytotoxic effects of these formulations on a range of cancerous and non-cancerous cell lines in in vitro assays, emphasizing the potential of both polyamine-1 and its liposomal formulation as promising therapeutic agents.

## 2. Materials and Methods

All reagents were obtained from commercial sources. Specifically, the phospholipidic compounds were purchased from Avanti Polar Lipids (Alabaster, AL, USA) or NOF (Tokyo, Japan). The dialysis membrane (Spectra/Por^®^6 Dyalisis, MWCO: 2000 Da) was provided by Fisher. ^1^H NMR and ^13^C NMR spectra were recorded on either a Bruker Advance 300 spectrometer operating at 300 MHz for ^1^H and at 75.4 MHz for ^13^C. Mass spectrometric analysis was performed under either ESI^+^ condition on a LCT Premier mass spectrometer. The UV/Vis spectrophotometer used was Cary 300. The human bladder cancer cell lines T24 (HTB-4) and 253J, the human breast cancer cell line MCF7 (HTB-22), the normal monkey kidney cell line VERO (CCL-81) and the mouse fibroblast cell line 3T3 (CCL-163) were purchased from the American Type Culture Collection (ATCC, Manassas, VA, USA).

### 2.1. Synthesis of 1,13-bis(1-Naphthylmethyl)-1,4,7,10,13,-Pentaazadecane Pentahydrochloride (Polyamine-1)

The synthesis of the compound has already been reported by some of us [29]. Briefly, a solution of 1-naphthaldehyde (2.82 g, 18 mmol) in 150 mL anhydrous ethanol was added dropwise to a solution of tetraethylenepentamine (1.63 g, 8.6 mmol) in anhydrous ethanol (150 mL) over 1 h and the mixture was stirred for 4 h at room temperature. NaBH_4_ (0.76 g, 20 mmol) was added, and the mixture was stirred for 2 h. The solvent was removed under reduced pressure and the resulting residue was treated with H_2_O (30 mL) and extracted with DCM (3 × 30 mL). The organic phase was dried over Na_2_SO_4_ and evaporated to dryness. The crude product was purified by column chromatography (silica; methanol/isopropilamine; 95:5 *v*/*v*) providing a yellow oil which was dissolved in anhydrous ethanol and precipitated, obtaining the product as its hydrochloride salt in 56% yield. ^1^H NMR (300 MHz, D_2_O): δ = 3.38–3.56 (m, 16H), 4.70 (s, 4H), 7.48–7.66 (m, 8H), 7.94–8.06 (m, 6H); ^13^C NMR (75.4 MHz, D_2_O): δ = 42.8, 43.5, 43.6, 44.5, 48.6, 122.4, 125.6, 125.6, 125.9, 126.7, 127.5, 129.1, 129.6, 130.8, 133.6; elemental analysis calc. (%) for C_30_H_44_Cl_5_N_5_: C, 55.57; H, 6.80; N, 10.74. Found: C, 55.8; H, 6.8; N, 10.9.

### 2.2. Synthesis of Diethylenetriamine Pentaacetic Acid bis-Stearylamide (DTPA-DSA)

The ligand was prepared using a protocol previously described by K. Kimpe et. al. with some modifications [39]. A solution of octadecylamine (1.35 g, 5 mmol) in 125 mL of DCM was added dropwise to a solution of diethylenetriaminepentaacetic dianhydride (0.96 g, 2.7 mmol) in 100 mL of DMF. The reaction mixture was stirred at 40 °C for 2 h. Then, it was let cold down to room temperature and stored overnight at 4 °C. The obtained white solid was filtered off, washed with acetone and vacuum dried overnight. Finally, the solid was crystallized from ethanol obtaining white crystals in 77% yield. ^1^H NMR (300 MHz, D_2_O): δ = 4.43–4.54 (d, 8H), 3.98–4.11 (d, 6H), 3.67 (s, 4H), 3.29–3.43 (t, 4H), 1.52–1.66 (s, 4H), 1.16–1.40 (s, 60H), 0.78–0.91 (t, 6H).

### 2.3. Synthesis of (Diethylenetriaminepentaacetic Acid)-bis(stearylamide) Gadolinium Salt (Gd-DTPA-DSA)

The complex was obtained following the same procedure reported by K. Kimpe et al. with minor changes [39]. A solution of GdCl_3_ (0.29 g, 1.1 mmol) in 1 mL of water was added dropwise to a solution of DTPA-DSA (0.89 mg, 1 mmol) in 30 mL of pyridine and the mixture was heated at 70 °C overnight. The solvents were removed under reduced pressure and the crude product was dissolved in boiling ethanol. After cooling it down to room temperature, the product was filtered off and vacuum dried overnight, obtaining a white solid in 15% yield. MS: *m*/*z* (%): 1073 ([M + Na]^+^).

### 2.4. Encapsulation of Polyamine-1 in Liposome Nanoparticles

The liposome nanoparticles containing the polyamine derivative were synthesized using lipid film hydration followed by sonication to produce unilamellar vesicles [40]. The lipid composition typically included POPC, DPPE-NBD and Gd-DTPA-DSA in a molar ratio of 83:2:5. In a standard synthesis procedure, the lipids were first dissolved in 5 mL of chloroform, and the solvent was then removed by rotary evaporation, resulting in a dry lipid film. This film was further dried under vacuum overnight. The dried lipid film was hydrated with an aqueous solution of polyamine-1 at 75 °C. To remove any free polyamine, the liposome suspension was subjected to thorough dialysis in milliQ water over a period of three days. The final liposome suspension was stored at 4 °C for subsequent studies.

### 2.5. Dynamic Light Scattering (DLS) and Z-Potential Measurements

The particle size and zeta potential of the liposomes were determined using a Malvern Mastersizer 2000, Malvern Instruments, Malvern, United Kingdom [41]. Samples were prepared by diluting the liposome suspension in deionized water and equilibrated at 25 °C before measurement. DLS measurements were performed in triplicate to ensure accuracy, and the results were averaged. The zeta potential was measured using the same equipment, with the samples equilibrated under the same conditions. Calibration of the equipment was performed according to the manufacturer’s instructions prior to measurements to ensure precision.

### 2.6. Inductively Coupled Plasma Mass Spectrometry (ICP-MS)

Gadolinium (Gd^3+^) content in the liposome nanoparticles was quantified using an ICP-MS-7900 system (Agilent Technologies, Santa Clara, CA, USA) [42]. Prior to analysis, the liposome nanoparticles were subjected to digestion with concentrated nitric acid overnight to ensure complete disruption and dissolution of the samples. After digestion, the samples were diluted to appropriate concentrations for ICP-MS analysis. The equipment was calibrated with standard solutions of Gd^3+^ to ensure accuracy and precision in the measurement of gadolinium content.

### 2.7. Electronic Microscopy

The morphology of the liposomes was analyzed using both Transmission Electron Microscopy (TEM) and Cryogenic Scanning Electron Microscopy (cryo-SEM) [43]. For TEM, a diluted liposome suspension was deposited onto a sample grid, allowed to air-dry and then stained with 3% phosphotungstic acid to enhance contrast. In cryo-SEM, a diluted liposome suspension was rapidly frozen in liquid nitrogen under vacuum to preserve its structure. Prior to imaging, the frozen samples were fractured and coated with a thin layer of gold to improve conductivity and image quality.

### 2.8. In Vitro Magnetic Resonance Imaging (MRI) Relaxivity Measurements and Phantom Study

The diagnostic capability of the liposomes was evaluated using MRI. Measurements were conducted on a 3T MR scanner (Achieva, Philips Medical Systems, Best, The Netherlands). The longitudinal relaxation time (T1) of the liposomes at varying gadolinium (Gd) concentrations was assessed using a gradient echo pulse sequence with the following parameters: field of view of 0.96 × 0.96 × 3 mm, flip angle of 60°, matrix size of 184 × 152 × 152 pixel and a repetition time (TR) of 100 ms and echo time (TE) of 3.2 ms. The longitudinal relaxivity (r1) in mM^−1^ s^−1^ was calculated from the slope of the linear regression of 1/T1 relaxation time versus Gd concentration (mM), as determined by ICP-MS [44].

### 2.9. Cell Culture Conditions

T24 and 253J transitional-cell human bladder carcinoma cells, MCF-7 human breast cancer cells, along with non-cancerous cells, including Vero green monkey kidney cells and 3T3 mouse fibroblasts were cultured at 37 °C in a 5% CO_2_ atmosphere. T24 and 253J cells were maintained in McCoy’s 5A medium, MCF-7 cells and Vero green monkey kidney cells were cultured in Eagle’s minimum essential medium (EMEM) while 3T3 mouse fibroblasts were cultured in Dulbecco’s Modified Eagle Medium (DMEM). All media were supplemented with 10% fetal bovine serum (FBS), 2 mM L-glutamine, 100 U/mL penicillin and 100 μg/mL streptomycin [45].

### 2.10. Cell Viability Assays

Cell viability was evaluated using the 3-(4,5-dimethylthiazol-2-yl)-2,5-diphenyltetrazolium bromide (MTT) assay. Cells were seeded at 2.5 × 10^4^ cells/mL in 96-well plates and treated with free polyamine-1, liposomes with encapsulated polyamine-1 and empty liposomes at concentrations of 1, 3, 6 and 12 μM for 48 h. The MTT assay measures the activity of mitochondrial dehydrogenases in viable cells, which reduce MTT to purple formazan crystals that are insoluble in water [46]. After treatment, the culture medium was removed, and 100 μL of MTT solution (5 mg/mL in PBS) was added to each well. The cells were then incubated for 4 h at 37 °C. The formazan crystals were dissolved by adding 100 μL of dimethyl sulfoxide (DMSO), and the absorbance of the resulting solutions was measured at 570 nm using a spectrophotometer, with a blank containing MTT and DMSO in a 1:1 ratio. The results were expressed as the percentage of MTT reduction, with the absorbance of control cells considered as 100%. Statistical evaluation of the results was performed and plotted using GraphPad version 5.0b (GraphPad Software Inc., San Diego, CA, USA). Unless specifically stated the data are represented as the mean ± S.E.M of the results obtained from three independent experiments [47].

## 3. Results

### 3.1. Synthesis and Liposome Preparation

Our strategy for the preparation of liposome nanoparticles was based on previously reported methods for producing unilamellar vesicles via sonication [48]. In this process, we mixed the phospholipids: 1-palmitoyl-2-oleoyl-sn-glycero-3-phosphocholine (POPC), 1,2-dipalmitoyl-sn-glycero-3-phosphoethanolamine-N-7-nitro-2-1,3-benzoxadiazol-4-yl (DPPE-NBD), gadolinium diethylenetriaminepentaacetic acid-bis(stearylamide) and a surfactant (Tween 80), in a molar ratio of 80/2/5/13. The lipid mixture was dissolved in a 1:1 chloroform/methanol solution (5 mL) and evaporated under nitrogen flux to yield a thin film, which was subsequently rehydrated. Hydration involved adding 2 mL of a water solution containing 6.51 mg of polyamine-1, with the mixture incubated at 65 °C and subjected to frequent vortex mixing. Finally, we sonicated the mixture twice for 15 min at 70 W at a 90% duty cycle.

**Figure 1:**
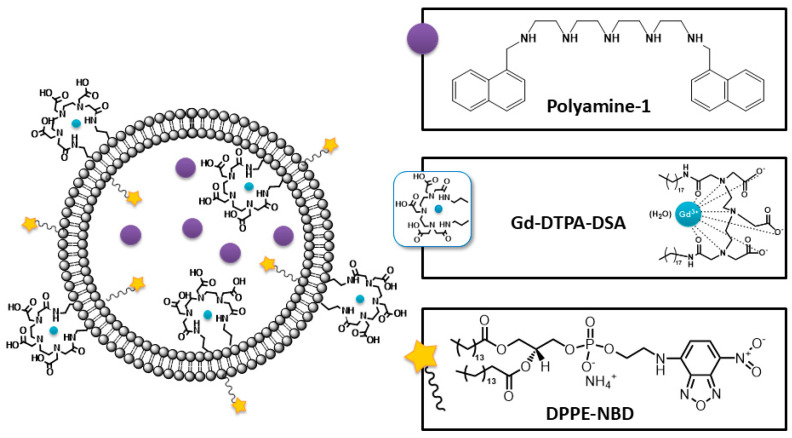
Schematic multimodal imaging loaded liposomes.

The liposomes were functionalised with a gadolinium complex (Gd-DTPA-DSA) and a fluorescent phospholipid (DPPE-NBD) (Figure 1) enabling them to also function as contrast agents for magnetic resonance imaging (MRI) and fluorescent probes for confocal microscopy.

### 3.2. Characterization of Liposomes

DLS analysis of liposomes revealed a monomodal size distribution with an average diameter of 130 ± 34 nm, indicating that the liposomes were uniformly sized. The polydispersity index (PDI) was measured at 0.345, suggesting relatively narrow size distribution and good homogeneity among the liposomes. To assess the stability of the liposomes in suspension, the zeta potential was determined to be −4.82 mV.

The size of the liposomes is a critical factor influencing their pharmacokinetics and therapeutic efficacy. The DLS analysis indicated that our liposomes have a size that is strategically advantageous for drug-delivery applications. Research has shown that liposomes smaller than 100 nm in diameter interact less with plasma proteins, avoid uptake by the reticuloendothelial system (RES) and have a longer half-life in the bloodstream, which enhances their ability to accumulate passively at tumor sites. However, while smaller liposomes benefit from these characteristics, they also have reduced drug-storage capacities [49]. Conversely, larger liposomes are more rapidly cleared from the bloodstream and are more readily taken up by the RES. By choosing a size of approximately 130 nm, our liposomes strike a balance between extended circulation time and sufficient drug-loading capacity, potentially optimizing their performance as effective delivery vehicles for targeted therapies [50].

The relatively low negative zeta potential (−4.82 mV) indicates that the liposomes have a modest electrostatic repulsion between particles. While this value is lower than the typically high zeta potentials associated with more stable nanoparticle systems, it still provides some degree of stability in suspension. Generally, a higher absolute zeta potential (above ±30 mV) is desired for stronger electrostatic stabilization, which helps prevent particle aggregation and improves dispersion. Despite this lower zeta potential, the liposomes have demonstrated notable stability, remaining in suspension over several months. This indicates that the liposomes maintain their dispersion and do not aggregate significantly over time. Therefore, the liposomes can still be effectively utilized as drug-delivery systems, with their current formulation proving suitable for extended stability and practical use in therapeutic applications [51].

**Figure 2:**
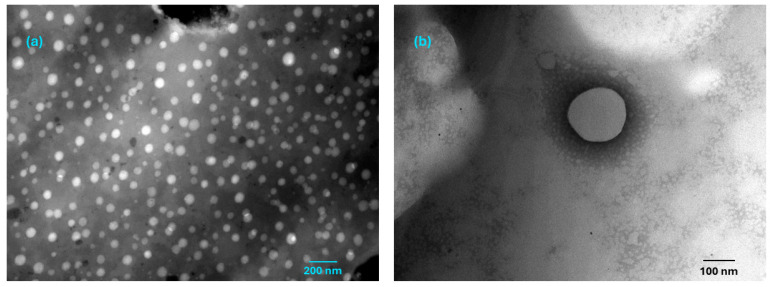
(**a**) TEM image of loaded liposomes prepared by sonication. (**b**) Isolated unilamellar spherical vesicle.

**Figure 3:**
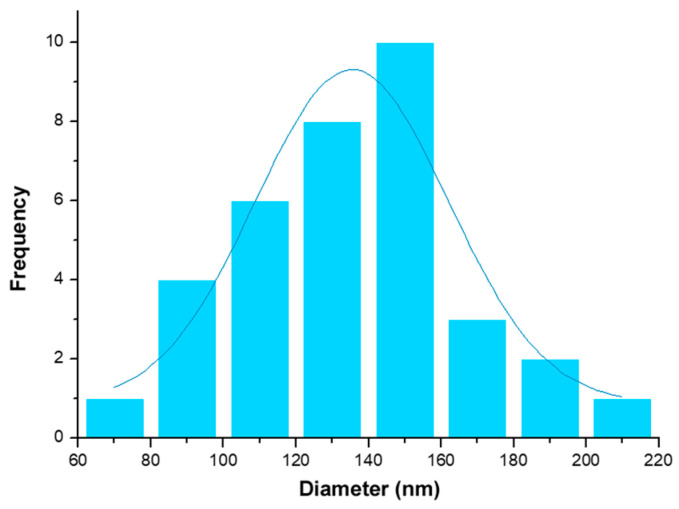
Histogram size distribution of the prepared liposomes.

Transmission electron microscopy (TEM) was used to determine the particle size, vesicle shape and lamellarity of liposomes. TEM images reveal the presence of unilamellar, structures with a spherical shape consisting of a lipid bilayer surrounding an aqueous core (Figure 2). The liposome diameter was typically in the range of 100–150 nm, which is consistent with the average diameter detected by DLS. A typical histogram of the size distribution is shown in Figure 3. The mean diameter of the liposomes prepared in this study was estimated to be approximately 130 nm.

**Figure 4:**
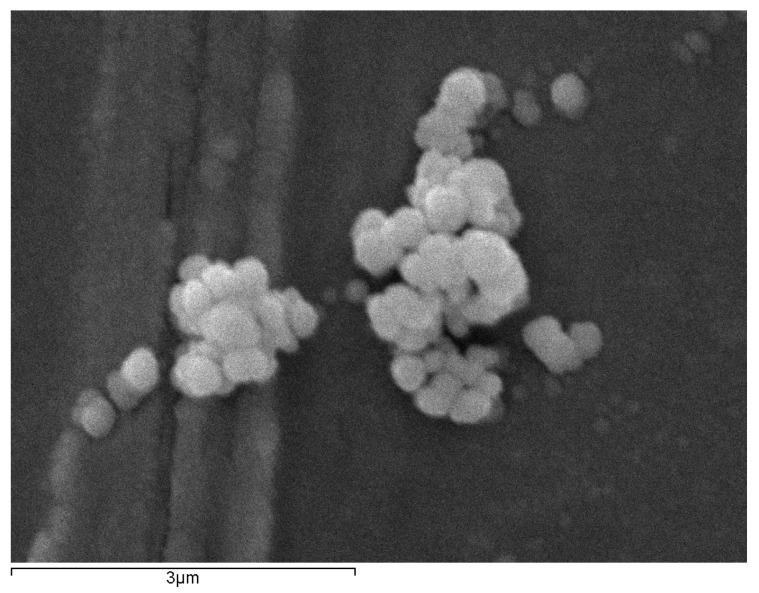
Cryo-SEM showing the spherical shape of unilamellar liposomes.

We also performed Cryo-Scanning Electron Microscopy (Cryo-SEM) on the liposomes prepared in this work to obtain morphological and partial 3D information about the shape of the vesicles. By rapid freezing, the Cryo-SEM technique can preserve the native structure of the liposomes. Figure 4 clearly shows that the shape of our liposomes was spherical under scanning electron microscopy, confirming the data obtained by TEM. The Cryo-SEM images also reveal a high degree of regularity and uniformity in the liposome structure, with consistent spherical shapes observed across different samples. Importantly, no structural irregularities were detected, further validating the stability and homogeneity of the liposome preparation.

### 3.3. Quantification of Encapsulation Efficiency

The concentration of the encapsulated compound within the liposomes directly influences the cytotoxicity of the nanoparticles. Therefore, quantification of this parameter is of crucial importance. The method used to determine the amount of encapsulated compound depends on the nature and physical properties of the cargo molecule. In our study, we quantified the amount of polyamine-1 encapsulated in the liposomes using UV/Vis spectroscopy.

A standard calibration curve was generated using several solutions of polyamine-1 at varying concentrations, with measurements based on the absorption maximum of the naphthalene group. The concentration of polyamine-1 within the liposomes was calculated by measuring the absorbance at 282 nm in three replicates and interpolating these values on the standard calibration curve (see Figures S1 and S2, and Tables S1 and S2 in the ESI†). The concentration of polyamine-1 encapsulated in the liposomes was found to be 1.01 ± 0.02 mM. With this concentration and the known amount of polyamine used during synthesis, we calculated the encapsulation efficiency.

The encapsulation efficiency was determined using the following equation, which represents the ratio of the encapsulated compound to the total compound used in the synthesis (Equation (1)) [52]:

(1) $$EE=\frac{\left[encapsulated\;compound\right]}{\left[encapsulated\;compound\;input\right]}\times 100$$

The encapsulation efficiency of polyamine-1 in the liposomes was calculated to be 30.3%. This efficiency is comparable to other compounds encapsulated in similar liposomal systems, which typically show an encapsulation efficiency ranging from 20% to 50% depending on the formulation and preparation method [53].

### 3.4. Cytotoxic Activity

The cytotoxicity of the liposome-encapsulated polyamine-1, free polyamine-1 and empty liposomes (without the compound) was evaluated across multiple cell lines to assess their potential as effective drug-delivery systems. The cell lines selected for this study included MCF7 (breast cancer), T24 and 253J (bladder cancer), 3T3 (mouse fibroblasts) and Vero cells (non-cancerous African green monkey kidney cells). These cell lines represent a range of cancer types and a non-cancerous control, providing a comprehensive evaluation of the formulation efficacy and selectivity.

**Figure 5:**
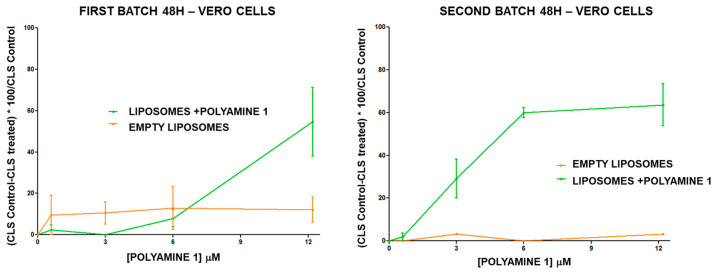
Percentage of cell death relative to the total number of cells in the control group (untreated) after 48 h of treatment. Results are shown for two different batches of liposomes: liposomes with encapsulated polyamine-1 and empty liposomes.

Figure 5 shows the percentage of dead cells relative to the total number of cells in the untreated control group after 48 h of treatment with two different batches of liposomes in Vero cells (a non-cancerous cell line). The results indicate that liposomes containing encapsulated polyamine-1 result in a higher percentage of cell death compared to empty liposomes, suggesting enhanced cytotoxicity. This observation highlights the therapeutic potential of the encapsulated polyamine-1. These findings support the effectiveness of the formulation and its possible utility in delivering therapeutic agents to target cells.

**Figure 6:**
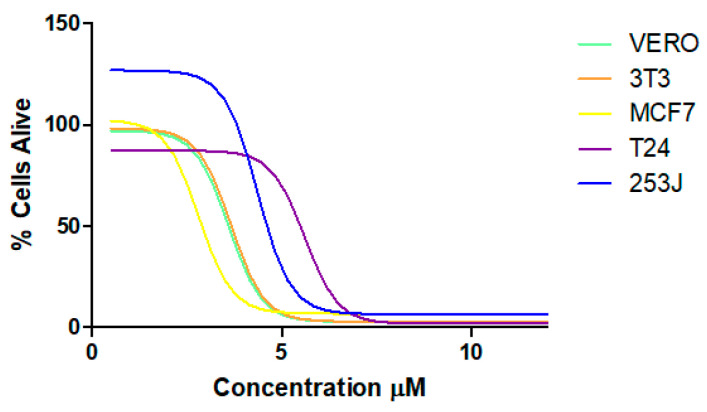
Percentage of live cells as a function of increasing concentration of therapeutic liposomes containing polyamine-1. The IC_50_ values are derived from the data for each cell line, representing the concentration at which cell viability is reduced by 50%.

To quantify the efficacy of liposome-encapsulated polyamine-1 across different cell lines, the IC_50_ values were determined. The IC_50_ represents the concentration of the compound required to inhibit cell viability by 50%. Figure 6 shows the percentage of live cells as the concentration of therapeutic liposomes increases, providing the IC_50_ values for all tested cell lines. At a concentration of 10 μM, the percentage of live cells is nearly zero across all cell lines, indicating potent cytotoxicity of the liposome-encapsulated polyamine-1.

The IC_50_ values varied between cell lines, reflecting the different sensitivities of each cancer type to the liposome-encapsulated polyamine-1. The IC_50_ values for each cell line are as follows: Vero cells: 3.6 µM; 3T3 cells: 3.6 µM; MCF7 cells: 2.8 µM; T24 cells: 5.6 µM; and 253J cells: 4.3 µM.

MCF7 cells are the most sensitive to the liposome-encapsulated polyamine-1. This suggests that the formulation is particularly effective against breast cancer cells. Conversely, T24 cells exhibit the greatest resistance to the therapeutic liposomes, as indicated by their highest IC_50_ value among the cancer cell lines. The IC_50_ value of Vero cells is comparable to that of 3T3 cells, indicating moderate sensitivity to the therapeutic liposomes in both cases. This suggests that while the liposomes are more effective against cancer cells, they also exhibit some level of toxicity toward non-cancerous cells.

To further evaluate the therapeutic potential of polyamine-1, we conducted cytotoxicity assays using the free form of the compound. This approach aimed to compare its efficacy directly against the liposome-encapsulated version and to assess its potency across various cancer and non-cancer cell lines. The IC_50_ values for free polyamine-1 were as follows: Vero cells: 0.10 µM; 3T3 cells: 0.30 µM; MCF-7 cells: 0.14 µM; T24 cells: 0.16 µM; and 253J cells: 0.23 µM.

These results reveal that free polyamine-1 has significantly lower IC_50_ values compared to the liposome-encapsulated form, indicating greater potency across all cell lines tested. Specifically, the IC_50_ value of 0.1041 µM for Vero cells indicates that free polyamine-1 is highly effective, even against non-cancerous cells, showing a level of potency comparable to that observed in MCF7 breast cancer cells. T24 and 253J bladder cancer cells exhibit moderate sensitivity to free polyamine-1, and among the cell lines tested, 3T3 mouse fibroblasts are the most resistant, indicating a lower level of sensitivity to the free polyamine-1.

Therefore, encapsulation of polyamine-1 in liposomes appears to enhance the therapeutic action of the compound specifically on tumor cells, while providing some degree of protection to non-tumor cells, compared to free polyamine-1. This encapsulation strategy appears to improve the selectivity of the treatment, effectively targeting cancer cells while reducing toxicity to non-cancer cells.

Moreover, both free and liposome-encapsulated polyamine-1 were screened at a concentration of 10 µM against the different cancer cell lines to evaluate their relative effectiveness and therapeutic potential. The goal of this study was to assess how each formulation impacts cell viability across different cancer types and to determine if encapsulation enhances the therapeutic efficacy of polyamine-1 while potentially reducing off-target effects.

**Figure 7:**
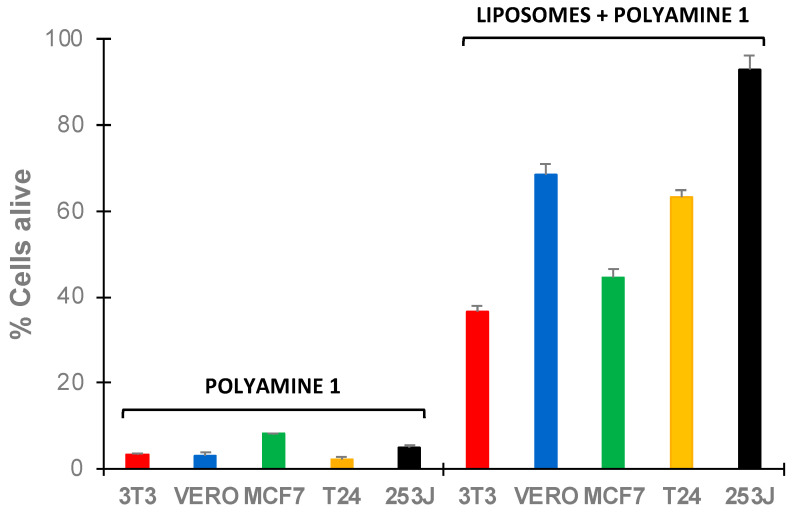
Percentage of live cells at a concentration of 10 mM for both free polyamine-1 and liposome-encapsulated polyamine-1 across various cell lines. The data are represented as the mean ± S.E.M of three determinations.

Figure 7 shows the percentage of live cells at a concentration of 10 µM for both free polyamine-1 and liposome-encapsulated polyamine-1. The data reveal that free polyamine-1 consistently exhibits greater cytotoxicity compared to its liposome-encapsulated form, with the percentage of live cells never exceeding 10% in any of the tested cell lines. Encapsulating the therapeutic compound in liposomes reduces its toxicity not only towards cancer cells but also towards non-cancerous cells, resulting in a survival rate of 68% in Vero cell lines. The most sensitive to the liposome-encapsulated polyamine-1 are the 3T3 cells (mouse fibroblasts), which show a survival rate of only 37%.

### 3.5. Imaging Studies

The search for a high relaxivity is a key feature for new contrast agents. In this regard, there are several strategies that can be employed to increase the relaxivity. In our case, we chose liposomes because of their slow tumbling (*τ*_R_), their easily tunable size and their flexibility to allow incorporation of hydrophilic and hydrophobic substances. In addition, these carriers benefit from a large number of gadolinium molecules that can be incorporated into their bilayer, resulting in an amplification of the relaxivity.

**Figure 8:**
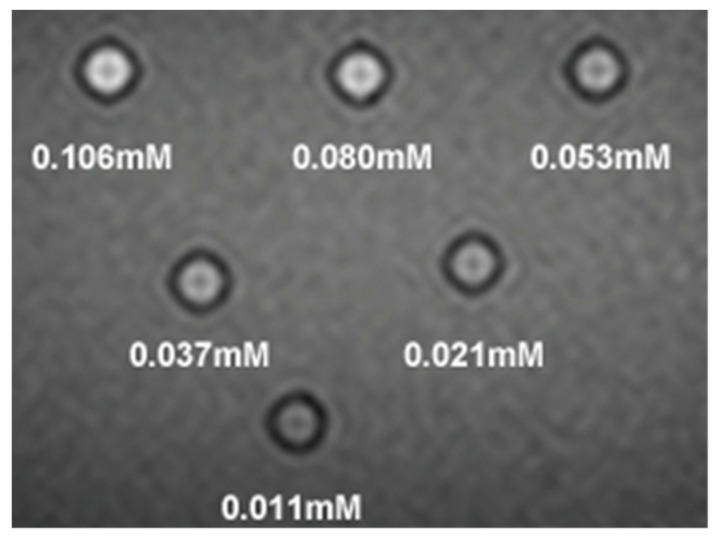
T1-weighted MR images at different concentrations of gadolinium.

An increase in signal intensity is observed on T_1_-weighted MR images of a phantom measured at 3 Tesla consisting of solutions containing increasing concentrations of the gadolinium loaded liposomes in water. The relaxivity measured on these conditions yielded a value of 2.62 mM^−1^s^−1^ (see Figure 8), which is lower than the r_1_ of free Gd-DTPA (3.9 mM^−1^s^−1^ measured at 8T) [54] and other liposomal formulations (4.7 and 4.5 mM^−1^s^−1^ measured at 3T) [55]. However, to fully determine the potential of these liposomes as contrast agents, it would be necessary to evaluate other crucial parameters such as circulation time, biodistribution, excretion and clearance rates, specificity for target tissues, toxicity, in vivo stability. While the r_1_ value is slightly lower, our encapsulation method offers nanoparticles with efficient gadolinium loading on the liposomal surface. Moreover, this encapsulation technique, which enables the incorporation of polyamines in liposomes, enhances their diagnostic utility in imaging applications and their therapeutic action.

## 4. Conclusions

In this study, we successfully developed a method for encapsulating polyamine-1 into liposome-based nanoparticles. Given the scarcity of publications on the encapsulation of polyamines in liposomal systems, this achievement is particularly noteworthy. Polyamine-1 previously demonstrated chemotherapeutic potential, largely attributed to its ability to intercalate DNA. A key advancement of this work lies in the evaluation of its antitumor activity, now tested against both cancerous and non-cancerous cell lines, expanding our understanding of its selective cytotoxicity.

The synthetized liposomes were thoroughly characterized, with parameters such as shape, size, polydispersity index and zeta potential being determined. The encapsulation efficiency was found to be within the typical range for these liposomal systems, confirming the viability of the formulation.

The encapsulation of polyamine-1 in liposomes significantly reduced its cytotoxicity compared to the free compound, resulting in an increase in the survival percentage of non-cancerous Vero cells to 68%. Despite the reduced toxicity to non-cancerous cells, liposomal formulation of polyamine-1 remained effective against cancer cells, demonstrating notable selectivity and a pronounced therapeutic effect across various cancer cell lines. This decrease in off-target toxicity, while maintaining therapeutic efficacy, is a key advantage for clinical applications.

Imaging studies using T1-weighted MR imaging revealed that gadolinium-loaded liposomes produced a significant increase in signal intensity, with a measured relaxivity value of 2.62 mM^−1^s^−1^. This amplification of relaxivity underscores the potential of these liposomes as effective contrast agents in MRI, enhancing their diagnostic utility. This dual functionality in therapeutic action and diagnostic capability highlights the potential for theranostic applications, combining the possibility of treatment with real-time monitoring.

In summary, our findings not only demonstrate that liposome-encapsulated polyamine-1 is a promising candidate for targeted cancer therapy but also introduce an advanced system capable of combining therapeutic delivery with MRI-based diagnostics. Future work could focus on optimizing the liposomal formulations to further improve encapsulation efficiency and enhance the selectivity and bioavailability of the nanoparticles. Additionally, combining polyamine-1 with other antitumor agents could provide synergistic effects, potentially improving therapeutic outcomes.

## Data Availability

All experimental data generated or analyzed during this study are included in the article.

## Supplementary Materials

The following supporting information can be downloaded at: https://www.mdpi.com/article/10.3390/biom14111347/s1, Figure S1: UV-Vis absorption spectra of Polyamine **1** solutions with different concentrations; Table S1: Data of absorbance and concentration of Polyamine **1** dilutions; Figure S2: Calibration curve obtained from the data of absorbance and concentration of Polyamine 1 dilutions extracted from Table S1; Table S2: Concentration of samples (liposomes with encapsulated Polyamine **1**) calculated by interpolation of the calibration curve.
