# Supplementary material for: An Effective Liposome-Based Nanodelivery System for Naphthalene Derivative Polyamines with Antitumor Activity
Source: Biomolecules. 2024 Oct 23;14(11):1347. doi: 10.3390/biom14111347 (PMC11591986; doi:10.3390/biom14111347)
Supplement: Supplementary file 1 [file biomolecules-14-01347-s001.zip › biomolecules-3249643-supplementary.pdf]

ELECTRONIC SUPPLEMENTARY INFORMATION

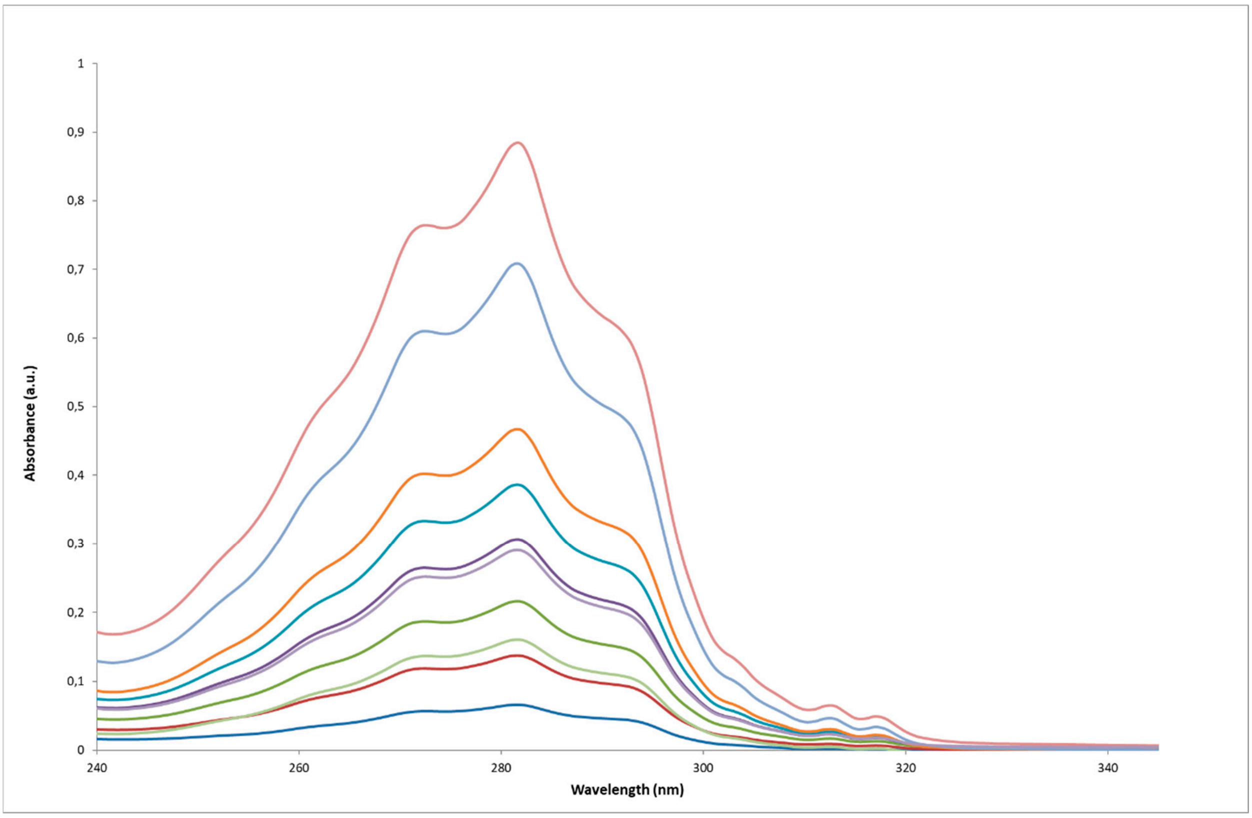

Figure S1 (ESI). UV-Vis absorption spectra of Polyamine **1** solutions with different concentrations.

Entry	$V_{aliquot}$ (µL)	[Polyamine 1] (M)	Absorbance at 282 nm
**1**	16	4.480E-06	0.061402798
**2**	20	5.601E-06	0.075109959
**3**	39	1.120E-05	0.14044952
**4**	51	1.456E-05	0.18023062
**5**	59	1.680E-05	0.20795918
**6**	63	1.792E-05	0.22195721
**7**	70	2.016E-05	0.24711847
**8**	78	2.240E-05	0.2767601
**9**	90	2.576E-05	0.31984997
**10**	117	3.360E-05	0.42175245
**11**	125	3.584E-05	0.45058346
**12**	156	4.480E-05	0.55647469

Table S1 (ESI). Data of absorbance and concentration of Polyamine **1** dilutions.

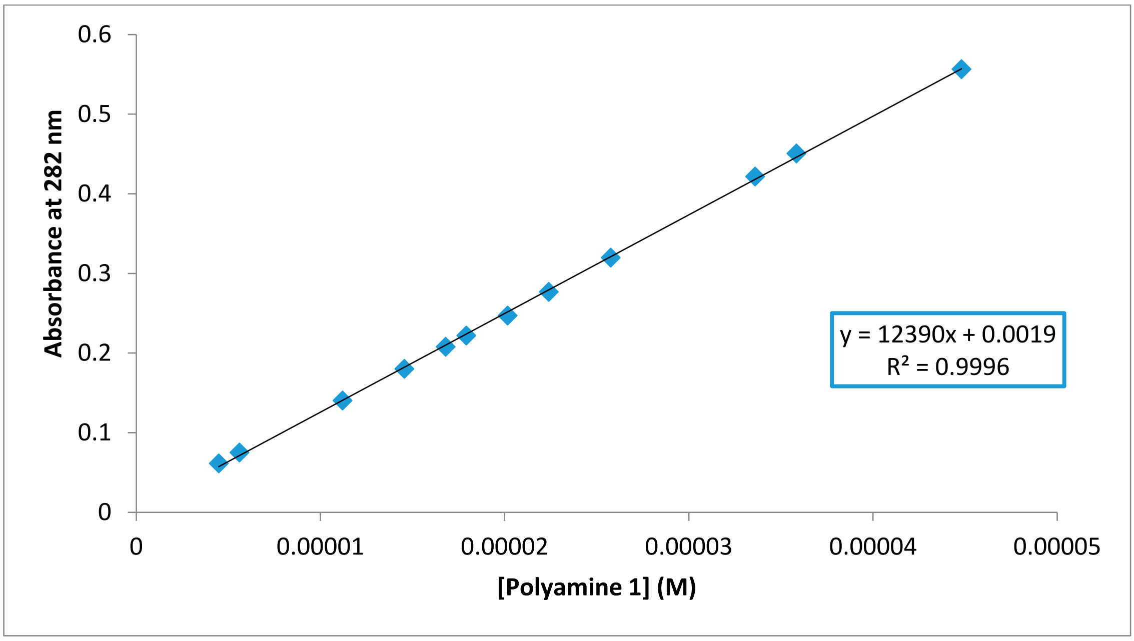

Figure S2 (ESI). Calibration curve obtained from the data of absorbance and concentration of Polyamine **1** dilutions extracted from Table 1.

Sample	Absorbance (282 nm)	[Polyamine 1]$_{sample}$ (mM)
**S1**	0.18579149	0.989461891
**S2**	0.19071865	1.01597337
**S3**	0.19434166	1.035467633
**Mean concentration**		**1.01 ± 0.02**

Table S2 (ESI). Concentration of samples (liposomes with encapsulated Polyamine **1**) calculated by interpolation of the calibration curve.
